# Supplementary material for: Expanded iOn switch toolkit enables flexible clonal labeling and dynamic imaging in model and non-model animals
Source: Commun Biol. 2026 Mar 24;9:654. doi: 10.1038/s42003-026-09907-1 (PMC13171893; doi:10.1038/s42003-026-09907-1)
Supplement: Supplementary file 1 — Supplementary Information [file 42003_2026_9907_MOESM1_ESM.pdf]

Expanded iOn switch Toolkit Enables Flexible Clonal Labeling and Dynamic Imaging in Model and Non-Model Animals

Authors

Zi Chao Ngiam[1,2 †], Kyosuke Wada[3,4 †], Jun Hatakeyama[5], Yuki Y. Yamauchi[6], Takuya Kaneko[7,8], Pauline Rouillard[6], Haruka Sato[5], Masahiko Hibi[7], Ikuo K. Suzuki[6], Carina Hanashima[1,2], Chiaki Ohtaka-Maruyama[3], Takuma Kumamoto[3*]

Supplementary information

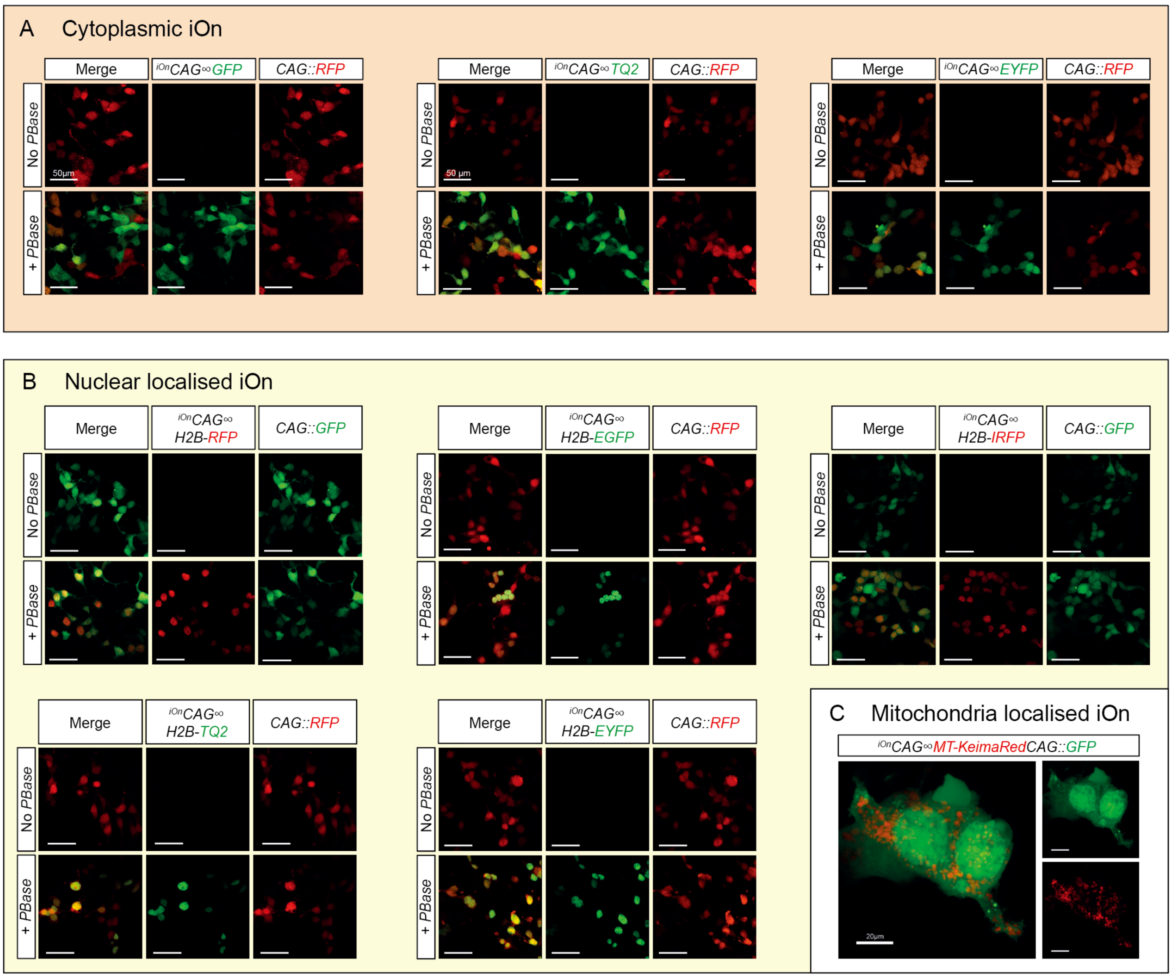

Fig. S1 Validation of newly generated iOn switch fluorescent and subcellular targeting variants in HEK293 cells

(A) Representative images of HEK293 cells transfected with newly constructed cytoplasmic iOn switch vectors, including ^{iOn}*CAG∞GFP*, -*Turquoise2 (TQ2)*, and -*EYFP*. (B) Nuclear-localized iOn switch variants, including H2B-tagged *RFP*, *EGFP*, *TQ2*, *EYFP*, and *IRFP*, demonstrate successful targeting to the nucleus. All constructs were validated three days post-transfection. (C) Expression of mitochondrial-targeted variant ^{iOn}*CAG ∞KeimaRed*, confirming proper subcellular localization.

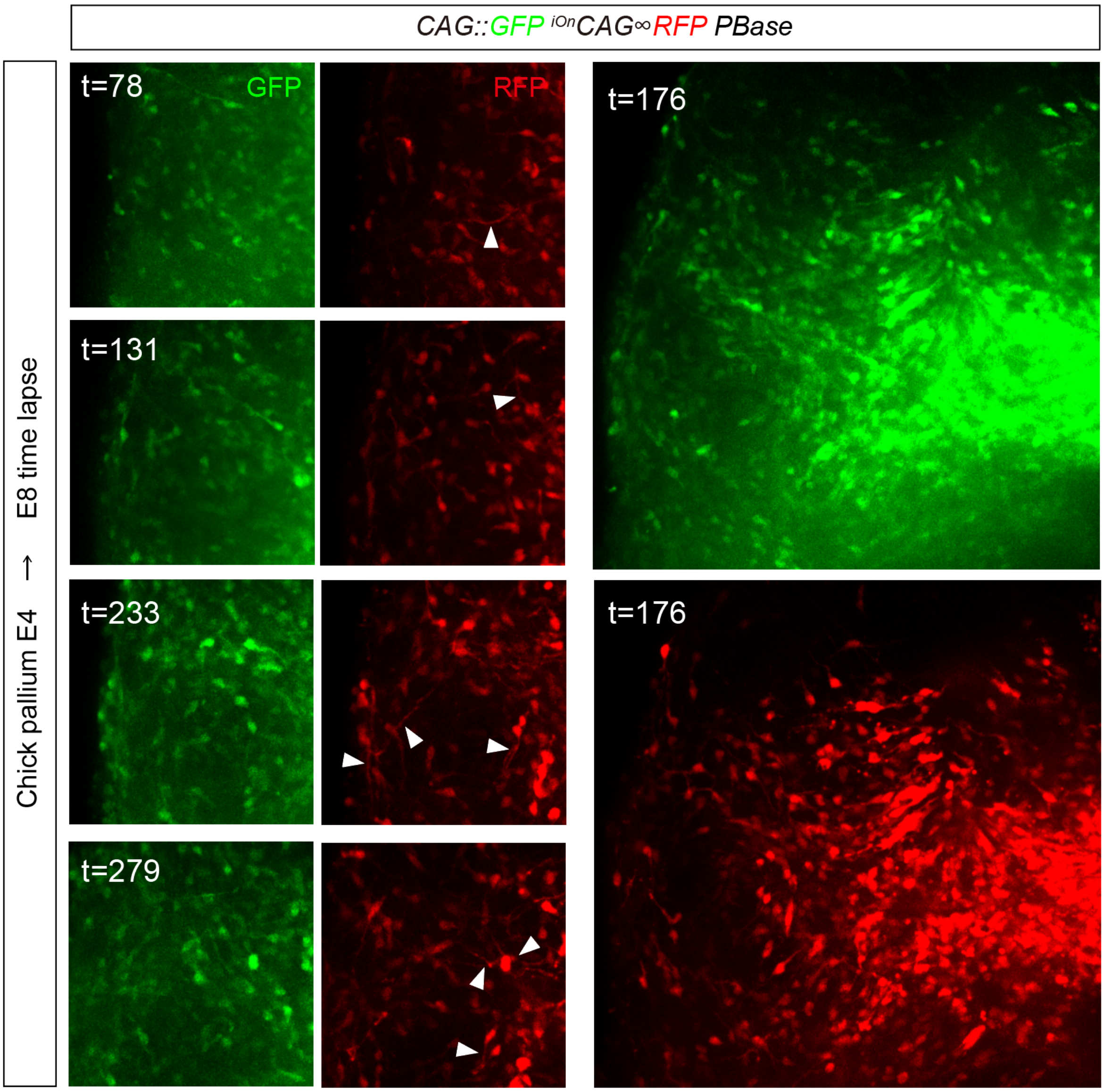

Fig. S2 Stable iOn-RFP expression supports consistent live imaging in chick cortex

Chick embryonic brains were electroporated at E4 with *CAG::GFP* and ^{iOn}*CAG∞RFP* plasmids, dissected at E6, and imaged over two days via time-lapse confocal microscopy. Time-lapse frames show representative expression of RFP and GFP across developmental timepoints. As observed in mouse (Fig. 4), *CAG::GFP* exhibits variable intensity, resulting in overexposure near the electroporation site and underexposure at more distal regions. In contrast, ^{iOn}*CAG∞RFP* expression remains stable and uniform, allowing reliable visualization of migrating cells without the need for dynamic exposure adjustment. Arrowheads mark neurite-like cellular processes highlighted by ^{iOn}*CAG ∞RFP* labeling.

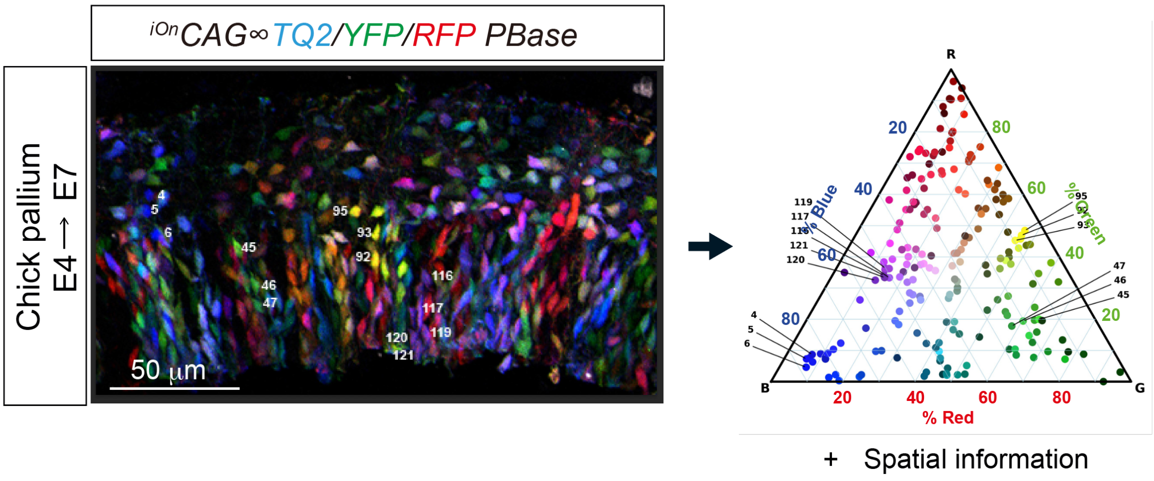

Fig. S3 Combining dense ternary plots with spatial information improves clone classification in densely labeled chick pallium

Chick pallium was electroporated at E4 with three-color iOn reporters (*^{iOn}CAG∞TQ2, YFP, and RFP*) together with *PBase* and analyzed at E7 (left). Individual cells were quantified for red/green/blue fluorescence, normalized to the total signal per cell, and plotted in ternary space (right; axes indicate the fraction of each fluorophore). In densely labeled samples, ternary position alone can be insufficient to separate neighboring clones with similar color composition. Therefore, we additionally incorporated spatial continuity (cell neighborhood/position in the tissue) when assigning cells to clones, enabling more conservative and accurate clone calls in high-density regions. Scale bar, 50 µm.

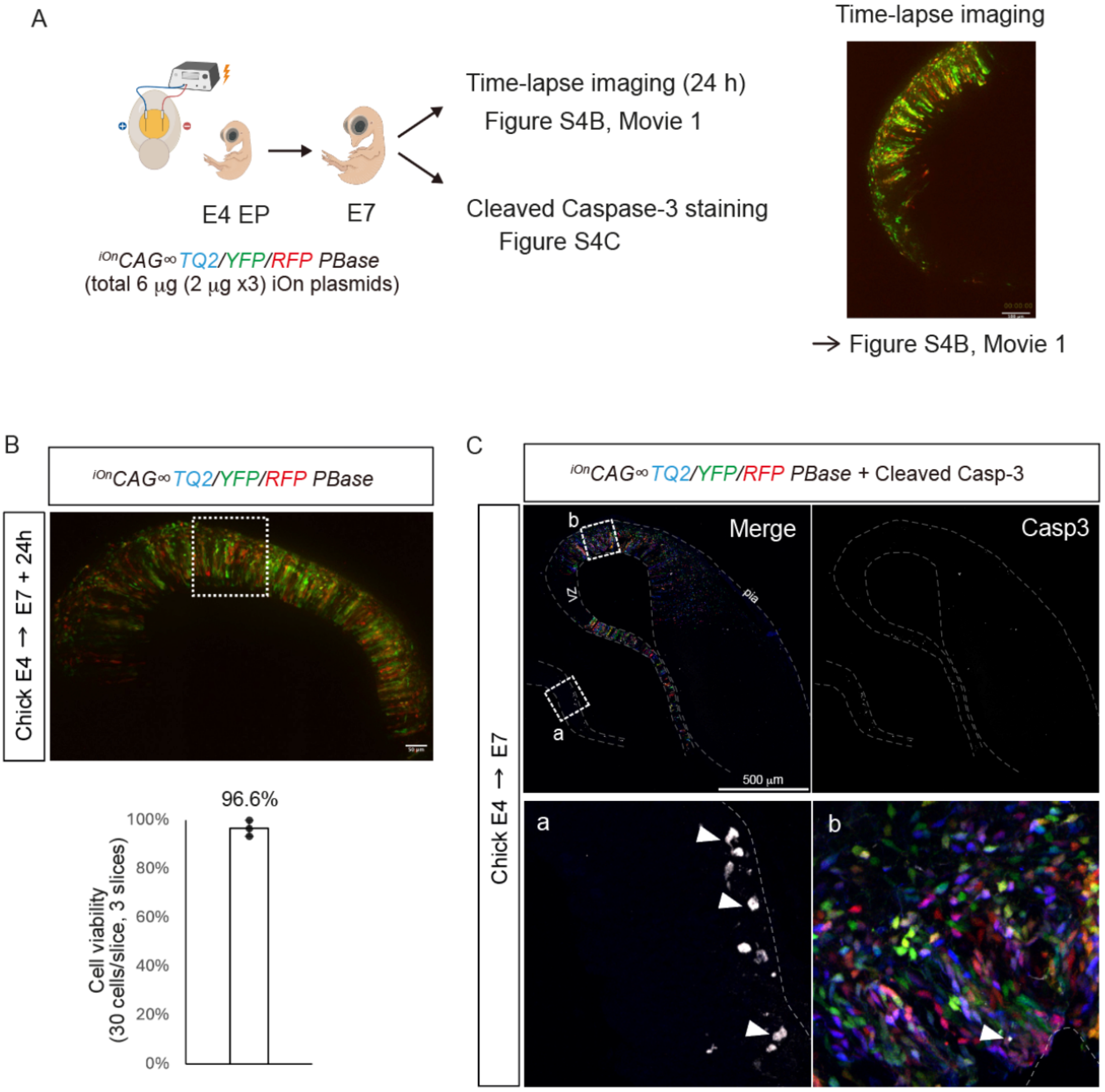

Fig. S4 The effect of cell toxicity by electroporating high-dose iOn plasmids

(A) Scheme of cell toxicity assay for this study. Created in BioRender. Kumamoto, T. (2026) https://BioRender.com/fxd2ol3. (B) Cell viability was quantified by time-lapse imaging. The white box marks the region shown in Movie 2. (C) Immunostaining against the activated form of Cleaved Caspase3 in chick brain sections 3 days after electroporation of the three iOn plasmids and PBase. Top: wide view of the brain section in merge channel (left) and Casp3 channel (right). Bottom: bottom images show enlarged view of boxed area, arrowheads point to Casp3-positive cells. Error bars indicate mean ± s.e.m.

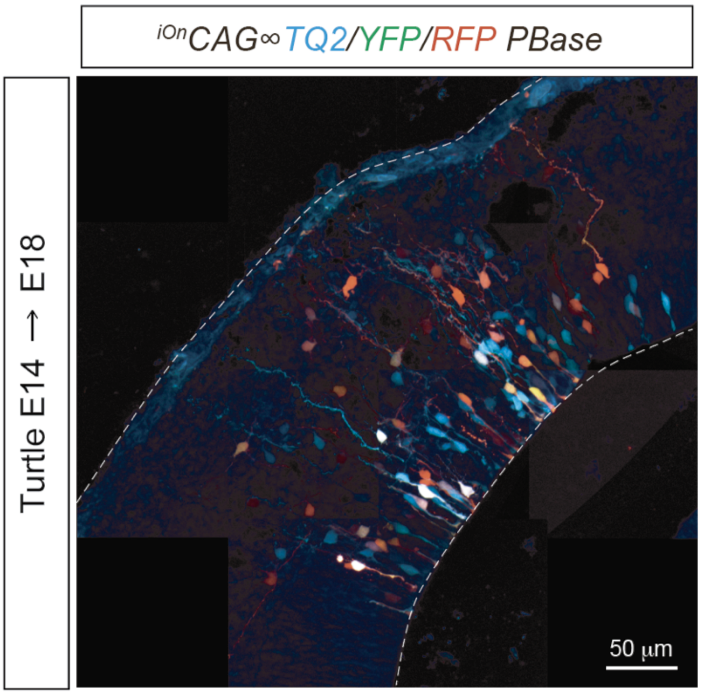

Fig. S5 Example images of guinea pig and turtle cortex electroporated with three-color iOn plasmids

Turtle pallium electroporated at E14 and analyzed at E18 with three-color iOn constructs (^{iOn}*CAG ∞ TQ2, -EYFP*, and *-RFP*).

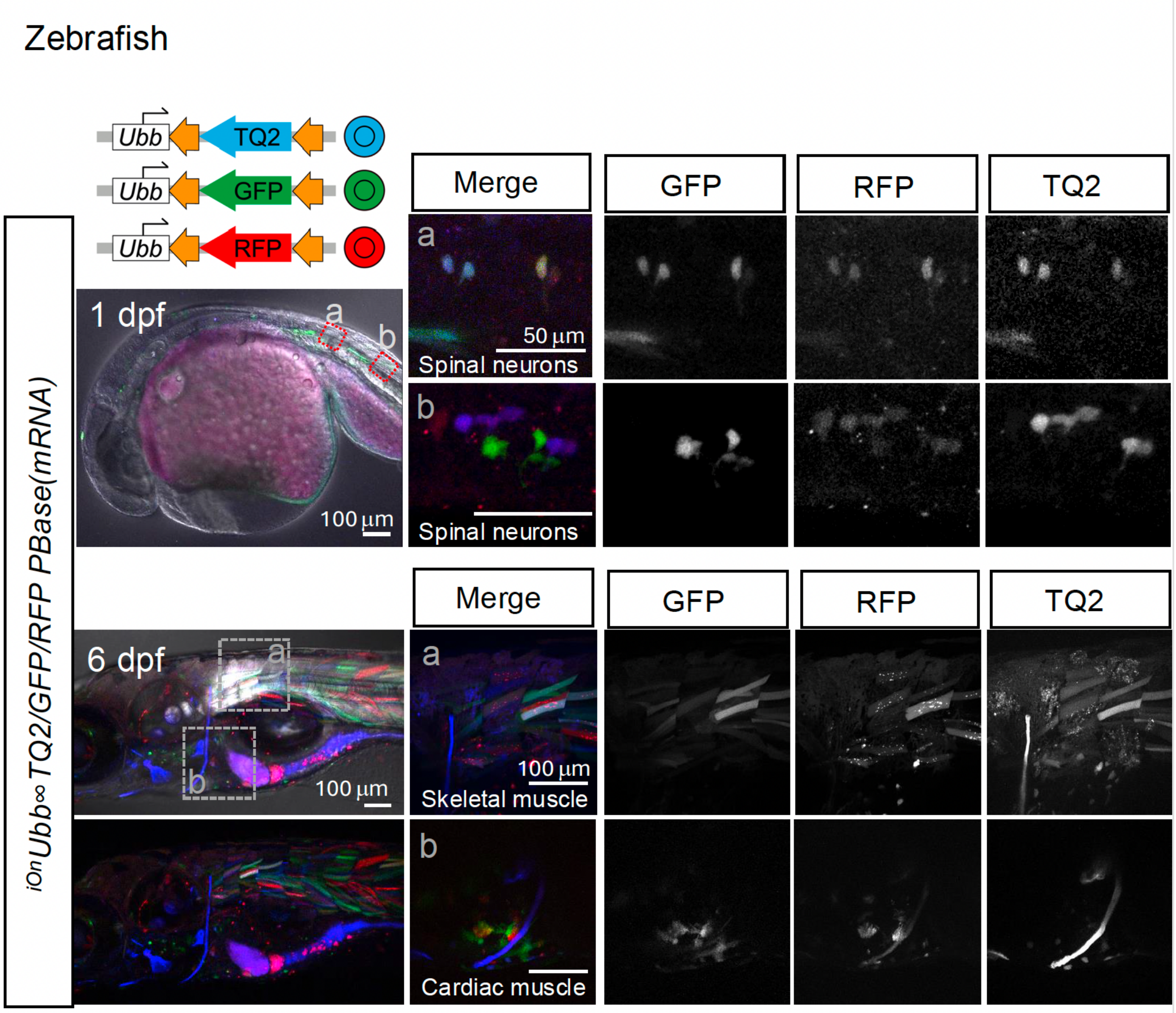

Fig. S6 Time-course expression of iOn switch in the zebrafish model

Zebrafish embryos were injected at 0 dpf (one cell stage) and analyzed at 1 dpf (top) and 6 dpf (bottom) with three-color iOn constructs (^{iOn}*Ubb* ∞*TQ2, -EGFP*, and *-RFP*). I A schematic of the iOn plasmids driven by the zebrafish Ubi promoter used in this study is shown at the top left. Panels (a) and (b) on the right show magnified views of the boxed regions.

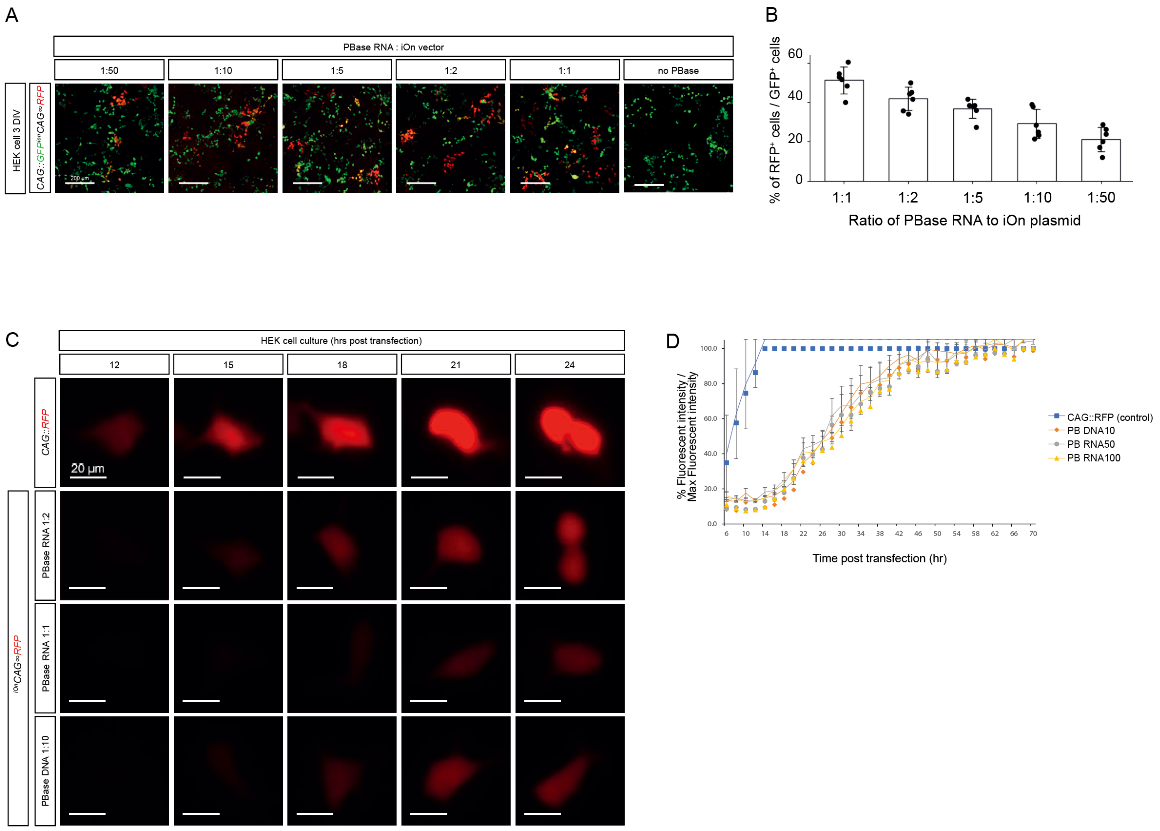

Fig. S7 Evaluation of mRNA-delivered PBase for accelerating iOn switch activation

(A) Representative images of HEK293 cells co-transfected with *CAG::GFP* and ^{iOn}*CAG∞RFP*, using varying amounts of *PBase* mRNA. (B) Quantification of transfection efficiency, expressed as the percentage of RFP$^+$ cells among GFP$^+$ cells. Only the 1:2 and 1:1 mRNA conditions approached the ~50% efficiency achieved with 1:10 *PBase* DNA. Error bars indicate n = 6 per condition (3 independent experimental repeats × 2 transfections). For each transfection, values from FoVs within a well were averaged to yield a single value. (C) Time-lapse imaging comparing the onset of RFP expression between ^{iOn}*CAG∞RFP* (1:10 *PBase* DNA, 1:2 and 1:1 *PBase* mRNA) and a *CAG::RFP* control. Representative images from 12 to 24 hours post-transfection. (D) Quantification of normalized fluorescence intensity over time across the different PBase delivery conditions. No significant difference in expression onset was observed, indicating that mRNA delivery does not accelerate iOn switch activation. Multiple cells per movie were averaged to yield a single movie-level value, and each movie was treated as one transfection replicate; n = 6 per condition (3 independent repeats × 2 transfections per condition). Error bars indicate mean ± s.e.m.

Note that the ratios reported above are mass ratios. Because *PBase* mRNA and plasmid DNA differ in length, the same mass ratio does not correspond to the same copy-number (molar) ratio. For reference, with iOn fixed at 100 ng (~0.029 pmol for the 5,223-bp plasmid), the *PBase* mRNA titration series tested here (100, 50, 20, 10, and 5 ng; 2,107 nt) corresponds to molar ratios (*PBase* mRNA:iOn DNA) of ~4.81:1, 2.41:1, 0.962:1 (~1:1), 0.481:1, and 0.241:1, respectively. A strict molar

match to the DNA–DNA condition (PBase:iOn ≈ 1:6.6 by molecules when using 0.2 µg *PBase* DNA and 1.0 µg iOn DNA) would require ~0.0315 µg (31.5 ng) *PBase* mRNA with 1.0 µg iOn DNA; this condition was not tested here.

Figure	Sample	Electroporation stage	Analysis stage	Voltage	No. of pulse	iOn plasmid	Conc.	HyPBase	Conc.	Control	Conc.
1A	HEK293	-	3 DIV	-	-	iOn CAG∞RFP	100ng	DNA	2ng	CAG::GFP	50ng
									10ng		
									20ng		
									50ng		
									100ng		
									-		
1C	Chick	E4	E7	15V	1	iOn CAG∞RFP	1ug	DNA	100ng	CAG::GFP	1ug
							500ng		50ng		
							100ng		10ng		
							50ng		5ng		
							10ng		1ng		
1F	Mouse	E14	E18	40V	4	iOn CAG∞RFP	1ug	DNA	100ng	CAG::GFP	1ug
							500ng		50ng		
							100ng		10ng		
							50ng		5ng		
							10ng		1ng		
2B	HEK293	-	3 DIV	-	-	iOn CAG∞TQ2	100ng	DNA	40ng		
						iOn CAG∞EYFP	100ng				
						iOn CAG∞RFP	100ng				
						iOn CAG∞iRFP	100ng				
2C	HEK293	-	3 DIV	-	-	iOn CAG∞RFP	100ng	DNA	30ng		
						iOn CAG∞H2B-iRFP	100ng				
						iOn CAG∞kras-GFP	100ng				
2D	HEK293	-	3 DIV	-	-	iOn CAG∞RFP	50ng	DNA	20ng		
						iOn CAG∞YFP	50ng				
						iOn CAG∞TQ2	50ng				
						iOn CAG∞H2B-RFP	17ng				
						iOn CAG∞H2B-YFP	17ng				
						iOn CAG∞H2B-TQ2	17ng				
3A	Chick	E4	E6	15V	1	iOn CAG∞TQ2	1ug	DNA	300ng	-	-
						iOn CAG∞EYFP	1ug				
						iOn CAG∞RFP	1ug				
3B	Mouse	E14	E16	40V	4	iOn CAG∞TQ2	400ng	DNA	120ng		
						iOn CAG∞EYFP	400ng				
						iOn CAG∞RFP	400ng				
4A	Turtle	E14	E23	15V	1	iOn CAG∞RFP	100ng	DNA	120ng		
						iOn CAG∞YFP	100ng				
						iOn CAG∞TQ2	100ng				
						iOn CAG∞H2B-RFP	100ng				
						iOn CAG∞H2B-YFP	100ng				
						iOn CAG∞H2B-TQ2	100ng				
4B	Rat	E15	P7			iOn CAG∞TQ2	1ug	DNA	300ng		
						iOn CAG∞EYFP	1ug				
						iOn CAG∞RFP	1ug				
4C	Guinea pig	E30	E52			iOn CAG∞TQ2	1ug	DNA	300ng		
						iOn CAG∞EYFP	1ug				
						iOn CAG∞RFP	1ug				
4D, S6	Zebrafish	0dpf	1-6dpf	microinjection		iOn Ubb∞TQ2	6.5pg	mRNA	30pg		
						iOn Ubb∞GFP	6.5pg				
						iOn Ubb∞RFP	6.5pg				
5	Mouse	E14	E16	35V	4	iOn CAG∞RFP	1ug	DNA	200ng	CAG::GFP	1ug
S1Ai	HEK293	-	3 DIV	-	-	iOn CAG∞EGFP	100ng	DNA	10ng	CAG::RFP	50ng
S1Aii	HEK293	-	3 DIV	-	-	iOn CAG∞TQ2					
S1Aiii	HEK293	-	3 DIV	-	-	iOn CAG∞EYFP					
S1B	HEK293	-	3 DIV	-	-	iOn CAG∞MT-KeimaRed					
S1Ci	HEK293	-	3 DIV	-	-	iOn CAG∞H2B-RFP					
S1Cii	HEK293	-	3 DIV	-	-	iOn CAG∞H2B-EGFP					
S1Ciii	HEK293	-	3 DIV	-	-	iOn CAG∞H2B-TQ2					
S1Civ	HEK293	-	3 DIV	-	-	iOn CAG∞H2B-EYFP					
S1Cv	HEK293	-	3 DIV	-	-	iOn CAG∞H2B-iRFP					
S2	Chick	E4	E8	15V	1	iOn CAG∞RFP	100ng	DNA	10ng	CAG::GFP	100ng
S7A	HEK293	-	3 DIV	-	-	iOn CAG∞RFP	100ng	RNA	2ng	CAG::GFP	50ng
									10ng		
									20ng		
									50ng		
									100ng		
									-		
S7C	HEK293	-	3 DIV	-	-	iOn CAG∞RFP	100ng	RNA	50ng	CAG::GFP	50ng
									100ng		
								DNA	20ng		
								-	-		

Table. S1 Summary of experimental conditions

Details of electroporation conditions, developmental stages, plasmid combinations, and DNA concentrations used in each experiment. This table is intended as a reference for optimizing iOn system usage in various biological contexts and species.
