# Supplementary material for: Expanded iOn switch toolkit enables flexible clonal labeling and dynamic imaging in model and non-model animals
Source: Commun Biol. 2026 Mar 24;9:654. doi: 10.1038/s42003-026-09907-1 (PMC13171893; doi:10.1038/s42003-026-09907-1)
Supplement: Supplementary file 2 — Description of Additional Supplementary Files [file 42003_2026_9907_MOESM2_ESM.pdf]

Description of Additional Supplementary files

File name: Supplementary Data 1

Description: Source data for figure 1, 5 and figure S4B

File name: Supplementary Data 2

Description: Supplementary Data for Figure S7D

File name: Supplementary Movie 1

Description: Representative time-lapse imaging supporting key results.

File name: Supplementary Movie 2

Description: Additional time-lapse/3D renderings supporting the study.
